# Symmetry in the developmental stages of permanent human teeth: a comparative study between maxilla and mandible

**DOI:** 10.1007/s40368-024-00996-2

**Published:** 2025-01-30

**Authors:** G. Haghi Ashtiani, J. A. Davies, H. M. Liversidge

**Affiliations:** Institute of Dentistry, Barts and The London School of Medicine and Dentistry, Queen Mary University of London, Turner Street, London, E1 2AD UK

**Keywords:** Symmetry, Developing teeth, Tooth formation, Panoramic radiographs

## Abstract

**Objective:**

The aim of this study was to assess symmetry of developmental stage of permanent teeth between the left and right side of the jaw, as well as between the maxilla and the mandible.

**Methods:**

A sample of 150 panoramic radiographs of individuals aged 6–20 years (69 males, 81 females) were selected from an open-access radiographic collection (Maxwell Museum of Anthropology’s orthodontic collection, Albuquerque, USA). All developing immature permanent teeth (*n* = 489) were scored by the first author using Moorrees and Demirjian tooth stages. Symmetry of developing teeth was assessed between the left and right sides of the jaw, as well as between the maxilla and the mandible using McNemar test with *p* < 0.05 considered significant.

**Results:**

No significant differences were found comparing left and right sides within the maxilla (*n* = 489), (McNemar, *p* = 0.759 M, *p* = 0.736 D), or within the mandible (McNemar, *p* = 0.262 M, *p* = 0.707 D) using either tooth scoring method. Percentage agreement for individual teeth between left and right sides was least for third molars. Significant differences were observed comparing maxillary and mandibular teeth for both tooth scoring methods (*n* = 978), (McNemar, *p* = 0.00 M, *p* < 0.001 D). Percentage agreement for individual teeth between the maxilla and mandible was least for incisors.

**Conclusion:**

In the present sample, differences in tooth formation were not significantly different in left and right side of the jaw while a significant difference (*p* = 0.00 M, *p* < 0.001 D) was observed between maxilla and mandible.

## Introduction

Tooth formation refers to the progressive stages of development within the alveolar bone, leading to mineralization and root formation, which is distinct from tooth eruption, or the process by which a tooth moves from the bone into a functional position within the oral cavity (Marks 1995). Dental age can be estimated from the age of eruption or the developmental stage of permanent teeth. However, tooth formation, which is less influenced by local factors and external variation than eruption timing, offers a more stable reference (Mattila and Haavikko 1969; Gleiser and Hunt 1955). The age of eruption of permanent teeth can be assessed directly by intraoral examination or indirectly on radiographs (Leurs et al. 2005). Dental radiography has been utilized in age estimation methods since 1982. Assessing dental findings through radiography serve as a significant information source in forensic odonatological age determination (Schmeling et al. 2001; Cameriere et al. 2006). It is important to highlight that panoramic radiographs offer a minimally invasive radiological method for estimating age, requiring only one image to capture the whole dentition. Additionally, these radiographs allow visualization of other bone structures like the mandible, condyles, coronoids, nasal fossa, and vertebrae (Vila-Blanco et al. 2023).

**Fig. 1:**
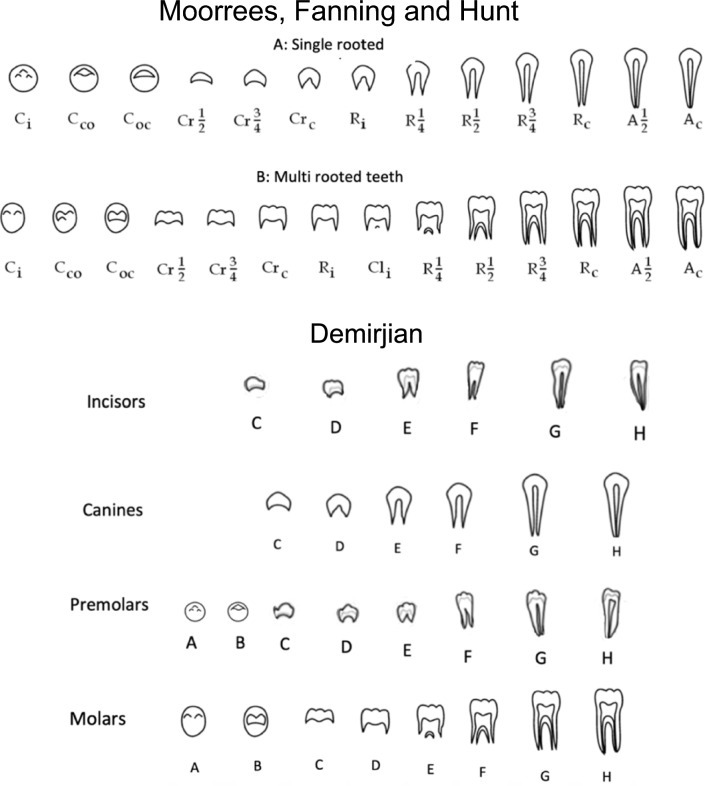
Stages of tooth development based on Moorrees, Fanning and Hunt and Demirjian methods. Moorrees, Fanning and Hunt: **A** upper drawings of single rooted teeth. **B** lower drawing for multi rooted teeth. C_i_ initial mineralisation, Cco coalescence of cusps, Coc cusp outline complete. Cr_1/2_ crown half completed with dentine formation, Cr_3/4_ crown three quarters completed, Crc crown completed with defined pulp roof, R_i_ initial root formation with diverge edges, Cl_i_ initial cleft formation, R_1/4_ root length less than crown length, R_1/2_ root length equals crown length, R_3/4_ three quarters of root length developed with diverge edge, Rc root length completed with parallel ends, A_1/2_ apex closed (root end converge) with wide PDL, Ac apex closed with normal PDL width. The eight stages (A–H) of tooth development from initial mineralisation through to root completion as developed by Demirjian. A Cusp tips are mineralized but have not yet coalesced, B Mineralized cusps are united so the mature coronal morphology is well-defined, C The crown is about 1/2 formed; the pulp chamber is evident and dentinal deposition is occurring, D Crown formation is complete to the dentino enamel junction. The pulp chamber has trapezoidal form, E Root length is less than the crown length, in molars formation of the inter-radicular bifurcation has begun. F Root length is at least as great as crown length. Roots have funnel-shaped endings, G Root walls are parallel, but apices remain open, H Apical ends of the roots are completely closed, and the periodontal membrane has a uniform width around the root

Evaluation based on the developmental stage of permanent teeth is more widely applicable because it is less affected by local factors and can be used for a wide range of ages (Yan et al. 2013). There are several methods for assessing the developmental stages of permanent teeth. A number of methods based on dental development have been used for age estimation. Two widely used approaches are the Moorrees, Fanning and Hunt (M) and Demirjian (D) methods (Moorrees et al. 1963a; Demirjian et al. 1973) (Fig. 1). Moorrees method follows Gleiser and Hunt (1955) who assessed longitudinal radiographs where the crown and root were divided into 13 and 14 crown, root and apical stages for single rooted and multiple rooted teeth, respectively (Moorrees et al. 1963a). The developing crown and root are divided into fractions of a quarter, half and three-quarter lengths. Demirjian et al. (1973) described a new tooth staging approach based on qualitative morphologic features rather than subjective fractions of the crown and root. Demirjian method classified tooth formation into eight stages from A to H, beginning at initial mineralisation and finishing at the radiographic closure of the root apex (Demirjian et al. 1973). Each tooth stage has three of four specific morphologic features that are described making identification of tooth stages easier and less subjective.

Previous studies that compared tooth eruption on the left and right sides used intraoral examination (Fulton and Price 1954; Nanda 1960; Sharma and Mittal 2001). Alternatively, studies focusing on the comparison of tooth formation on the left and right sides used intraoral examination, oblique X-rays, and orthopantomographs (Garn et al. 1958; Grøn 1962; Moorrees et al. 1963b; Hirano et al. 2009; Kuremoto et al. 2022). According to these studies, no significant differences exist between the left and the right side in the eruption of teeth (Fulton and Price 1954; Nanda 1960; Sharma and Mittal 2001) or in their formation (Garn et al. 1958; Grøn, 1962; Moorrees et al. 1963b). Garn et al. (1958) and Grøn (1962) focused mainly on the calcification stages of mandibular teeth, which provided early insights into bilateral symmetry but were limited in scope by primarily assessing mandibular rather than maxillary teeth (Garn et al. 1958; Grøn 1962). Moorrees et al. (1963a, b) expanded this research to include maxillary teeth, but these studies typically used single-stage methods and did not consistently account for subtle differences in developmental stages within each jaw (Moorrees et al. 1963b).

Most studies compare the age of eruption/tooth stage of left side and right side (Hirano et al. 2009; Kuremoto et al. 2022), except Grøn who expresses the frequency of asymmetry in root development (Grøn 1962).

Previous studies have consistently reported symmetry in the developmental stages of left and right teeth within both jaws (Fulton and Price 1954; Garn et al. 1958; Nanda 1960), reinforcing the reliability of using contralateral teeth as references in clinical and forensic contexts. While much of the research has focused primarily on mandibular teeth or did not separate findings by jaw or developmental stage, some studies have examined both maxillary and mandibular teeth, though gaps in the literature remain.

The application of widely recognised staging methods, such as Moorrees and Demirjian, allows for a more comprehensive understanding of developmental symmetry.

In addition to examining symmetry within each jaw, several studies have reported developmental differences between the maxilla and mandible. For instance, Van der Linden (2016) described distinct eruption sequences between the jaws, with mandibular canines typically erupting before premolars, whereas in the maxilla, premolars commonly precede canines (Van der Linden 2016). Additionally, mandibular anterior teeth often develop and erupt earlier than their maxillary counterparts, a sequence potentially influenced by structural and growth-related differences such as root length and jaw growth dynamics (Lam and Koudela 2010). These inter-jaw developmental differences underscore the importance of separate analyses of maxillary and mandibular teeth to accurately capture developmental patterns unique to each jaw.

In contrast to tooth formation, tooth eruption has demonstrated significant variation between the maxillary and mandibular arches, as well as between the left and right sides, with polymorphisms observed across populations (Smith and Garn 1987; Diamanti and Townsend 2003; Natarajan et al. 2018; Šindelářová and Broukal 2019). For example, Garn et al. (1958), Grøn (1962), and Moorrees et al. (1963b) have shown symmetry in calcification stages, although earlier studies often reported findings without the level of clarity seen in recent literature.

Most dental age reference data are based on mandibular teeth while limited data exists for maxillary teeth. To date, no recent studies assessed symmetry or asymmetry of tooth development and mainly focused on mandibular teeth. This paper attempts to extend previous work on symmetry in tooth formation.

The aim of this study was to assess symmetry of developmental stage of permanent teeth between the left and right side of the jaw, as well as between the maxilla and the mandible**,** highlighting new insights provided by this comparative analysis.

## Materials and methods

### Ethical approval

Ethical approval was not required for the online open access resource of the Maxwell Museum of Anthropology’s orthodontic collection, Albuquerque, USA.

### Sample

The sample used in this study comprised panoramic radiographs of 150 individuals aged 6 to 20 years (69 males, 81 females) obtained from the online open-access resource of the Maxwell Museum of Anthropology’s orthodontic collection, Albuquerque, USA. Individuals could identify with multiple options, allowing for a potentially multi-ethnic sample composition. The available ethnic and geographic categories in this collection include American Indian or Alaskan Native, Asian American, Black or African American, Hispanic or Latino, Native Hawaiian or other Pacific Islander, and White or European American**.**

The sample size was determined to ensure a representative distribution across the 6 to 20-year age range, allowing for sufficient statistical power in each subgroup. Sample size calculation was performed to ensure statistical power, though it is acknowledged that a larger sample size would provide more robust results.

The images were anonymized for chronological age and sex. The inclusion and exclusion criteria were designed to eliminate potential confounding factors such as dental anomalies or previous orthodontic treatments, ensuring the reliability of the findings. Inclusion criteria were the presence of all permanent teeth on both the left and right sides of the jaws. Exclusion criteria included any disturbances affecting normal dental development, presence of supernumerary teeth, hypodontia, and evidence of previous extraction of a permanent tooth. Table 1 shows the sample distribution according to sex and age categories.

**Table 1:** Distribution of the sample by age and sex

Age in years	Female (*n*)	Male (*n*)	Total (*n*)
6	7	4	11
7	5	3	8
8	7	4	11
9	4	6	10
10	4	5	9
11	1	0	1
12	4	5	9
13	6	3	9
14	8	5	13
15	7	12	19
16	2	8	10
17	7	3	10
18	8	2	10
19	5	5	10
20	6	4	10
Total	81	69	150

### Tooth staging methods

**Fig. 2:**
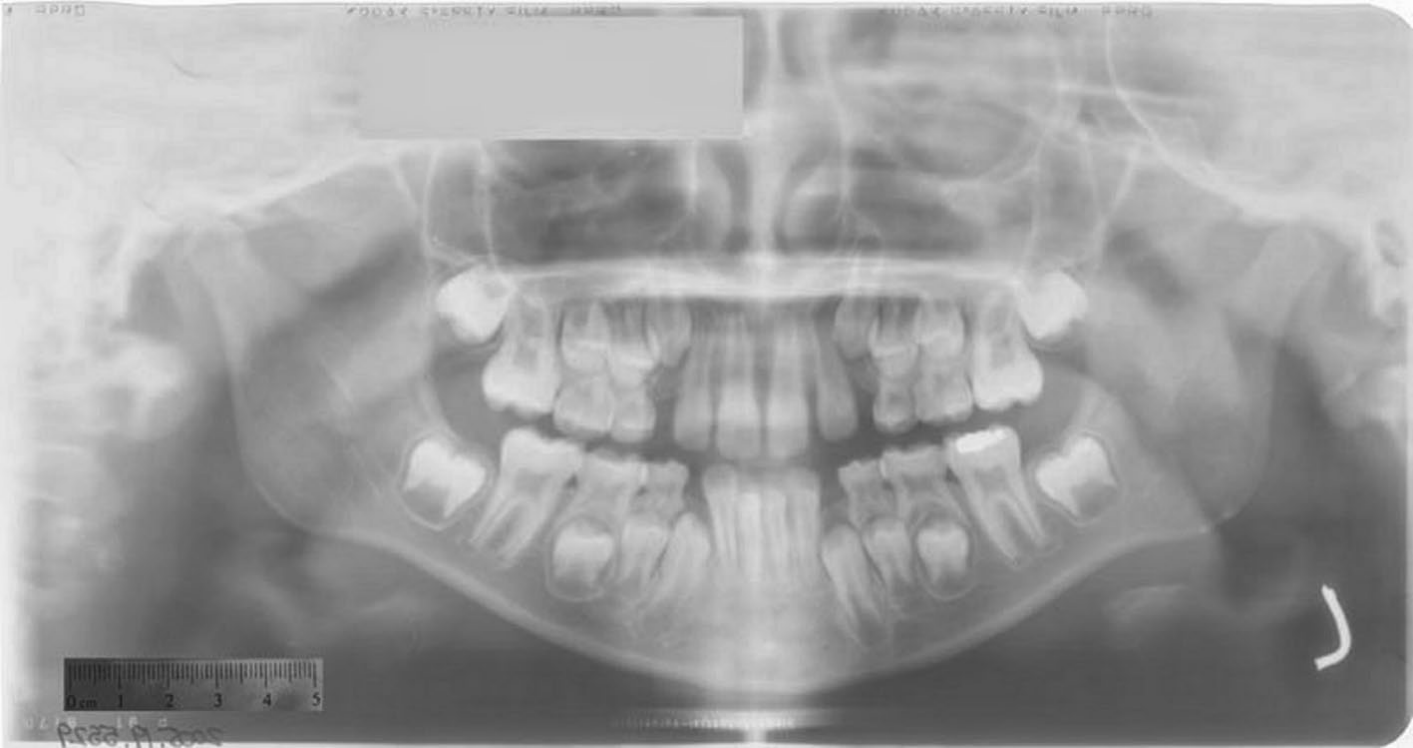
Panoramic Radiograph included in this study. In this, male maxillary incisors staged Rc (root length completed with parallel ends): Moorrees, G (Root walls are parallel): Demirjian, mandible incisors as Ac (apex closed with normal PDL width): Moorrees, H (Apical ends of the roots are completely closed): Demirjian, canines as R_1/4_ (root length less than crown length): Moorrees, E (Formation of the inter-radicular bifurcation has begun): Demirjian, first and second premolars as R_i_ (initial root formation with diverge edges): Moorrees, E (Formation of the inter-radicular bifurcation has begun): Demirjian, first molars as Rc (root length completed with parallel ends): Moorrees, G (Root walls are parallel): Demirjian and second molars as Cl_i_ (initial cleft formation): Moorrees & D (Crown complete): Demirjian

To minimize bias and ensure objectivity, a rigorous blinding process was implemented. The observers who scored the developmental stages of the teeth were blinded to the details of the radiographs, including the age and sex of the individuals. All the radiographs were anonymized and coded. All developing immature permanent teeth (*n* = 489) were scored using the Moorrees, Fanning and Hunt (M) and Demirjian (D) stages by the first author. Symmetry was assessed based on identical stages defined in the Moorrees and Demirjian methods. The criteria for scoring symmetry and asymmetry among right and left teeth in the same jaw, as well as those of teeth in opposing jaws, were based on these stages. An example of the scoring on a panoramic radiograph is provided in Fig. 2.

Tooth stage assessment was undertaken in the same order starting from the maxillary left side teeth, from the central incisor to the third molar, and then left side mandibular teeth were assessed in the same order. The same steps were repeated for maxilla and mandible teeth on the right side. Tooth stages were assigned numbers and recorded into a Microsoft Excel 2019 version 16.28 spreadsheet for non-parametric statistical analysis.

### Inter and intra-observer agreement

Inter and intra-observer agreement was assessed by having the first and second authors independently score 30 panoramic radiographs. This process was repeated after a 1 week interval. Weighted kappa values were then calculated to determine the levels of agreement between and within observers.

### Statistical analysis

Symmetry of developing teeth was assessed between the left and right sides of the jaw, as well as between the maxilla and the mandible using McNemar test with *p* < 0.05 considered significant (lack of symmetry). All statistics were performed using the Statistical Package for Social Science statistical software (version 26; SPSS Inc., Chicago, Illinois).

## Results

### Reliability

From 30 radiographs, Kappa values were 0.87 and 0.91 for inter-observer and intra-observer, respectively.

### Left/right tooth symmetry

**Fig. 3:**
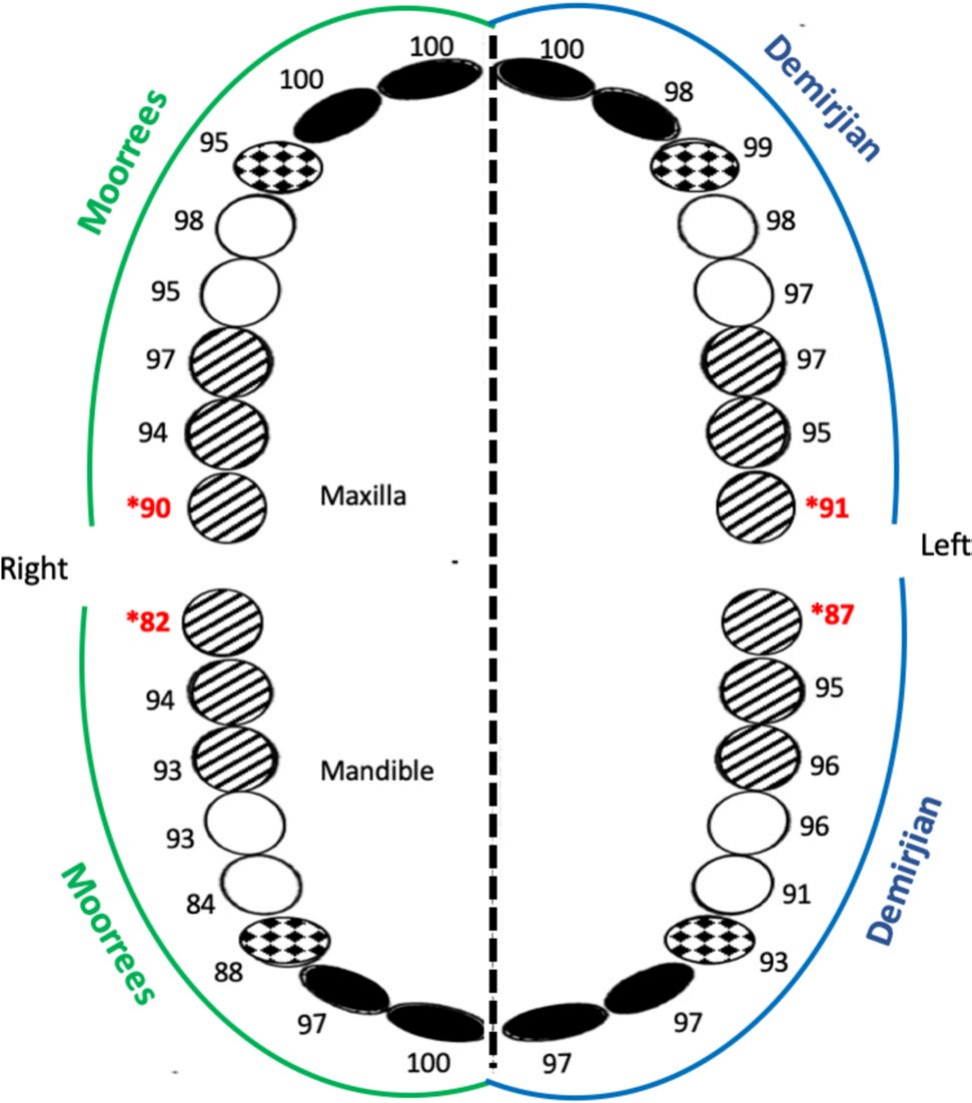
Percentage of agreement for individual teeth between left and right side of jaws using Moorrees & Demirjian methods. *Percentage of agreement was least for third molars. Incisors filled, canines stippled, premolars open, molars diagonal lines

Percentage of agreement for individual teeth between left and right sides of maxilla using Moorrees and Demirjian stages ranged 89.51–100% and 90.9–100%, respectively. This percentage for left and right sides of mandible was 81.81–100% for Moorrees and 90.9–100% for Demirjian stages. In both methods, percentage of agreement of was the highest for incisors and the lowest for third molars (Table 2 and Fig. 3). High percentage of agreement among left and right sides of both jaws demonstrates the symmetry in tooth development using Moorrees and Demirjian stages.

**Table 2:** Percentage of agreement for individual teeth between left and right side of jaws using Moorrees & Demirjian methods

Tooth	Number of teeth (*n*)	Left and right side of maxilla				Left and right side of mandible			
		Moorrees		Demirjian		Moorrees		Demirjian	
		L&R identical readings (*n*)	Symmetry of left and right side (%)	L&R identical readings (*n*)	Symmetry of left and right side (%)	L&R identical readings (*n*)	Symmetry of left and right side (%)	L&R identical readings (*n*)	Symmetry of left and right side (%)
M3	143	128	89.5	130	90.9	117	81.8	124	86.7
M2	62	58	93.5	59	95.1	58	93.5	59	95.1
M1	30	29	96.6	29	96.6	28	93.3	29	96.6
P2	58	55	94.8	56	96.5	54	93.1	56	96.5
P1	55	54	98.1	54	98.1	46	83.6	51	91
C	59	56	94.9	58	98.3	52	88.1	55	93.2
I2	41	41	100	40	97.5	40	97.5	40	97.5
I1	41	41	100	41	100	41	100	40	97.5
All	489	462	94.4	467	95.5	436	89.1	454	92.8

*L* Left, *R* Right

For analysis of symmetry, the difference in developmental stage was tested between the left and right teeth using Moorrees and Demirjian stages. No significant differences were found comparing left and right sides within the maxilla (*n* = 489), (McNemar, *p* = 0.759 M, *p* = 0.736 D), or within the mandible (McNemar, *p* = 0.262 M, *p* = 0.707 D) using either tooth scoring method (Table 4).

### Maxilla/mandible symmetry

**Fig. 4:**
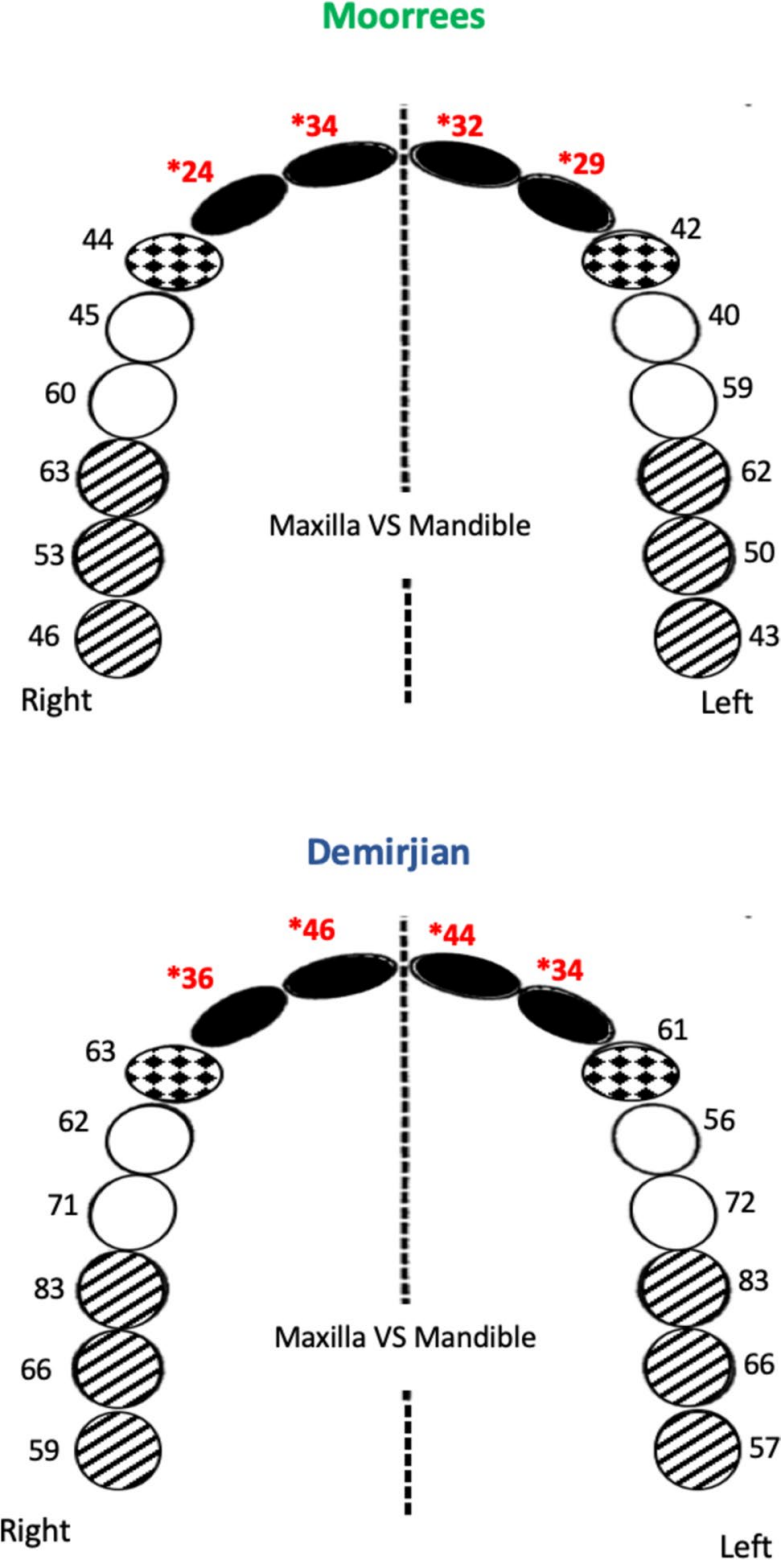
Percentage of agreement for individual teeth between maxilla and mandible using Moorrees & Demirjian methods. Incisors filled, canines stippled, premolars open, molars diagonal lines. *Percentage of agreement was least for incisors

Percentage of agreement for individual teeth between the maxilla and mandible’s left side using Moorrees and Demirjian stages was 29.26–62.06% and 34.14–83.33 respectively. This percentage between the maxilla and mandible’s right side was 24.39–63.33% for Moorrees and 36.58–83.33% for Demirjian stages. Lateral and central incisors had the lowest percentage of agreement however this percentage was the highest for first molars and second pre-molars using both methods (Table 3 and Fig. 4). When comparing maxilla and mandible, percentage of agreement was slightly higher in posterior teeth than anterior showing the asymmetry in tooth development using Moorrees and Demirjian stages.

**Table 3:** Percentage of agreement for individual teeth between maxilla and mandible using Moorrees & Demirjian methods

Tooth	Number of teeth (*n*)	Maxilla vs mandible				Maxilla vs mandible			
		Moorrees		Demirjian		Moorrees		Demirjian	
		U&L identical readings (*n*)	Symmetry of Maxilla & Mandible (%)	U&L identical readings (*n*)	Symmetry of Maxilla & Mandible (%)	U&L identical readings (*n*)	Symmetry of Maxilla & Mandible (%)	U&L identical readings (*n*)	Symmetry of Maxilla & Mandible (%)
M3	143	62	42.6	81	56.6	67	46	85	59.4
M2	62	31	50	41	66.1	33	53.2	41	66.1
M1	30	19	62	25	83.3	19	63.3	25	83.3
P2	58	34	58.6	42	72.4	35	60.3	41	70.6
P1	55	22	40	31	56.3	23	41.8	34	61.8
C	59	25	42.3	36	61	26	44	37	62.7
I2	41	12	29.2	14	34.1	10	24.3	15	36.5
I1	41	13	31.7	18	43.9	14	34.1	19	46.3
All	489	218	44.5	288	58.8	227	46.4	297	60.7

*U* Maxilla-Upper, *L* Mandible-Lower

For further analysis of symmetry, the difference in developmental stage was tested between the maxilla and mandible teeth using Moorrees and Demirjian stages. Significant differences were observed comparing maxillary and mandibular teeth for both tooth scoring methods (*n* = 978), (McNemar, *p* = 0.00 M, *p* < 0.001 D) (Table 4).

**Table 4:** Mcnemar test within and between jaws using Moorrees & Demirjian methods

	Total number of teeth (*n*)	*p* Moorrees	*p* Demirjian
Maxilla left vs right	489	0.759	0.736
Mandible left vs right	489	0.262	0.707
Maxilla vs mandible	978	0.00*	< 0.001*

*McNemar test with *p* < 0.05 considered significant (lack of symmetry)

## Discussion

This study assessed symmetry of developmental stage of permanent teeth between the left and right side of the jaw, as well as between the maxilla and the mandible using Moorrees and Demirjian methods. These results suggest symmetric development for left and right side in both the maxilla and the mandible while asymmetric development was observed between maxilla and mandible.

Percentage of agreement for individual teeth between the left and right side of the jaw, as well as between the maxilla was slightly higher for Demirjian method compared to Moorrees stages. This reflects more agreements and fewer differences in Demirjian method compared to Moorrees stages and may relate to the fewer number of tooth stages in Demirjian compared with Moorrees.

With weighted kappa values *κ* > 0.87, both the inter-agreement and intra-agreement can be classified as very good, indicating that the observers were well-calibrated.

Tooth formation is a continuous process and may be classified into stages. Different systems are available for classifying stages of tooth development. They differ with regard to number of stages and presentation and definition of each stage. Some classification systems are defined by changes in tooth and root form, e.g., the method developed by Moorrees, Fanning and Hunt and Demirjian. Therefore, two popular methods are used for this study.

Differences in the eruption of first molars between the right and left sides of the jaw have been identified (Hirano et al. 2009), while no significant difference in the growth rates of bilateral homonymous teeth at different developmental stages was observed (Kuremoto et al. 2022).

In the present study, we found symmetry in developmental stage of permanent teeth among left and right sides of both jaws. This suggests that the results for the left and right sides can be combined. Moreover, contralateral homologous tooth on one side can be used when it is difficult to identify the developmental stage of a tooth on the other side. Homologous refers to corresponding teeth on the left and right sides of the jaw, such as left and right first molars. The findings of no significant differences between left and right sides of both jaws align with previous research, which reported similar symmetry (Garn et al. 1958; Grøn 1962). This reinforces the concept that left and right developmental stages are comparable and can be used interchangeably in forensic and orthodontic evaluations. However, the research expands the understanding of this symmetry by using two well-established staging methods, providing a comprehensive analysis. By employing both the Moorrees and Demirjian methods, we add a new dimension to this established understanding, highlighting the consistency across different scoring systems.

From a clinical perspective, asymmetric development between the maxilla and mandible is expected as mandibular anterior teeth have shorter roots and tend to erupt earlier than the corresponding teeth in the maxilla (Lam and Koudela 2010). This is supported by the radiographic study of Anderson et al. (1976), which found that the mean age of attainment of the maxillary central incisor was 10.6 years, whereas the mandibular central incisor was 9.2 years in males (Anderson et al. 1976). Additionally, mandibular incisors take a shorter time to develop than maxillary incisors and are smaller in all dimensions compared to maxillary incisors (Nelson 2014). The study not only confirms these developmental asymmetries but also provides quantitative analysis using the Moorrees and Demirjian methods, contributing to understanding of these differences.

There is little evidence-based information available, and clinicians and forensic odontologists often assume symmetry in dental development. This study provides the necessary evidence, showing that the observed symmetry between the left and right sides in both jaws supports the use of contralateral teeth as reliable references in clinical and forensic evaluations. This finding enhances the applicability of dental age estimation methods. Furthermore, the significant asymmetry between maxillary and mandibular teeth underscores the need for differential analysis when assessing dental development stages, which has practical implications for orthodontic planning and forensic casework.

One limitation of the study is the demographic origin of the sample, primarily comprising individuals of European ancestry from the Maxwell Museum’s orthodontic collection. This collection includes self-reported demographic data where individuals could select multiple ethnic or geographic categories; however, the sample predominantly represents European ancestry, a factor that may limit the applicability of the findings to broader populations. Additional ethnic or geographic categories within this collection include American Indian or Alaskan Native, Asian American, Black or African American, Hispanic or Latino, and Native Hawaiian or other Pacific Islander. While this lack of diversity may limit the applicability of the findings to broader populations, it is important to consider the relevance of these demographic factors in modern society. The critical need for inclusive and diverse representation in clinical research has been emphasised to ensure that findings are applicable to all demographic groups (Flanagin et al. 2021). As the global population becomes increasingly diverse, understanding and addressing health disparities that may exist across different ethnic and geographic groups is essential. In light of these considerations, future studies should aim to include more diverse samples to enhance the generalisability of findings and ensure their relevance to modern society.

Another limitation of this study is the small sample size for each age group resulting from the limited availability of high-quality panoramic radiographs. Although the findings demonstrate statistically significant differences, a larger sample size would provide improved statistical power, enabling more robust subgroup analyses and enhancing the generalizability of the study.

The radiographs which were obtained from the online open access resource of the Maxwell Museum of Anthropology’s orthodontic collection, Albuquerque, USA, were originally wet film which have been digitised so a loss of quality is to be expected. Therefore, additional methods such as intraoral examination or oblique X-rays are needed to complement and confirm the findings of this study.

Future research should aim to address these limitations by including a larger and more diverse sample, to enhance the generalisability of the findings. Furthermore, using multiple sources of radiographic data could improve the robustness and accuracy of the results.

Assessing symmetry of developmental stage of permanent teeth between the left and right side of the jaw offers several key advantages including implication for forensic age estimation, the correlation between chronological and dental age and sequence of tooth eruption between left and right side of the jaws. Moreover, the extent of asymmetric development between the left and right side of the arch can indicate the presence of some problem, i.e. pathological lesion, such as cysts or tumors, which need to be determined and treated accordingly.

This study provides evidence of symmetry in the developmental stages of permanent teeth between the left and right sides of both jaws. These findings have practical implications, such as in the assessment of dental maturity where methods require seven mandibular teeth; if one tooth is unclear or missing, the contralateral tooth can be used. In forensic age estimation, reference data from one side of the mandible can be applied when only the other side is available. Additionally, fragments of the maxilla can be used to estimate age based on mandibular reference data.

The observed differences between maxillary and mandibular teeth contribute to a deeper understanding of dental growth patterns, which can inform clinical practices and forensic investigations, ultimately benefiting dental professionals and organizations.

## Conclusion

In the present sample and given the limitations described, differences in tooth formation were not significantly different in left and right side of the jaw while a significant difference was observed between maxilla and mandible, however, this should be confirmed using a larger sample and wider age range. This study contributes novel insights into the symmetry of dental development, with potential applications in improving age estimation methods and enhancing orthodontic treatment planning.

## Data Availability

The data supporting the findings of this study are included in this published article. Additional data can be provided upon request from the corresponding author.
